# Salt, aldosterone and extrarenal Na^+^ - sensitive responses in pregnancy

**DOI:** 10.1016/j.placenta.2017.01.100

**Published:** 2017-08

**Authors:** Paula Juliet Scaife, Markus Georg Mohaupt

**Affiliations:** a Division of Child Health, Obstetrics and Gynaecology, School of Medicine, 1st Floor Maternity Unit, City Hospital Nottingham, Nottingham NG5 1PB, United Kingdom; b Department of Clinical Research, University of Bern, Switzerland, 3Sonnenhof Hospital, Berne, Switzerland

**Keywords:** Sodium sensing, Placenta, Macrophages, Aldosterone, Pre-eclampsia

## Abstract

Outside of pregnancy excessive salt consumption is known to be harmful being linked to increased blood pressure and cardiovascular disease. However, pregnancy represents a major change to a woman's physiology resulting in an intimate adaptation to environmental conditions. It is now becoming apparent that salt is essential for a number of these changes during pregnancy including haematological, cardiac adaptations as well as directly influencing placental development and the uteroplacental immune environment. The present review discusses the important role that salt has during normal pregnancy and evidence will also be presented to show how the placenta may act as a salt sensing organ temporarily, yet substantially regulating maternal blood pressure.

## Highlights

• The placenta may function as an extrarenal regulator of maternal blood pressure.
• Na^+^handling in pregnancy is completely different to the non-pregnant situation.
• Na^+^may actually lower blood pressure in pregnancy affected with pre-eclampsia.
• Aldosterone is an important regulator of placental and fetal development.
• Na^+^ may compensate for aldosterone deficiency in pregnancy.

## 1 The physiological need for salt during pregnancy

The role of excessive dietary salt (sodium chloride, NaCl) in causing hypertension, cardiovascular disease and stroke is now well established [1]. However, sodium is also essential for healthy physiological function as it is required for the regulation of fluid levels, temperature and pH. The recommended level of dietary salt intake is 5 g per day, which equates to around 2000 mg of Na^+^ [2], however dietary salt intake is above this recommended daily amount in a majority of countries [3]. As blood pressure responses in a healthy population follows a Gaussian distribution [4], very low levels of salt intake may even stimulate pressure responses of the renin angiotensin II system (RAS) [5].

In pregnancy dietary salt intake seems to facilitate the numerous physiological changes that must occur to support the growth and development of the placenta and foetus. These changes affect every organ system in the body and begin shortly after conception [6] (see Table 1). For the majority of women these changes resolve following parturition with no long term residual effects. However, for certain pathological conditions, for example the hypertension present in pre-eclampsia, it may take much longer for pre-pregnancy physiologically normal levels to return.

**Table 1:** Physiological changes that occur during normal human pregnancy.

Body system	Physiological adaptation to pregnancy
Haematological	Plasma volume expansion occurs to around 50% of pre-pregnancy volume. However, red blood cell volume increases by only 20–30% resulting in a fall in haemoglobin concentration, haematocrit and red blood cell count [63], [64].
Cardiovascular	Large increase in cardiac output from early pregnancy onwards [65]. Peripheral vasodilation results in a fall in systemic vascular resistance which is compensated for by increased stroke volume and also (but to a lesser extent) an increase in heart rate [66].
Renal	There are increases in both renal blood flow by around 50% and glomerular filtration rate (GFR) by between 30 and 50% which results in an increase in the fractional excretion of protein by up to 300 mg/day. Increased renal blood flow leads to an increase in renal size of 1–1.5 cm [67], [68]. Activation of the renin-angiotensin system (RAS) leads to increased plasma levels of both renin and aldosterone and subsequent salt and water retention in the distal tubule and collecting duct. However there is dissociation between plasma concentrations of renin and aldosterone during pregnancy. Increases in aldosterone concentrations from pre-pregnancy values are proportionally greater than those of renin by the third trimester [7], [69].

Haematological changes are an important part of the maternal physiological response to pregnancy. Blood volume increases gradually during pregnancy by approximately thirty to fifty percent and this is proportional to the birthweight of the baby. The expansion in plasma volume is greater than the increase in erythrocyte cell number. The increase in plasma volume is crucial for maintaining circulating blood volume, blood pressure and uteroplacental perfusion during pregnancy. Plasma volume expansion is brought about by increased salt and thirst appetite combined with activation of the RAS. In close interaction, angiotensin II and to a large extent VEGF stimulate aldosterone and subsequent salt and water retention in the kidneys [7], [8]. Sodium retention occurs at a rate of approximately 2–6 mmol Na^+^ per day [9] resulting in increased total body sodium of around 1000 mEq [10] as plasma osmolality falls [11], [12] by 10 mosmol/kg below non-pregnant levels [11], [13]. Increased activity of the RAS is mediated by not only an increased production of renin by the kidneys but also renin production by the ovaries and the uteroplacental unit which produce an inactive precursor protein of renin during early pregnancy [14]. Yet, in contrast to non-pregnant women in pregnancy angiotensinogen secretion from the liver driven by placental production of oestrogens results in proportionally increased levels of aldosterone. Plasma levels of aldosterone correlate well with those of oestrogens and rise progressively during pregnancy [15]. Natriuresis still occurs despite the sodium retaining properties of aldosterone due to the potent antagonist actions of progesterone and the rise in glomerular filtration rate which allows excretion of excess sodium. Furthermore, progesterone acts to inhibit kaluresis thereby ensuring that potassium excretion is kept constant throughout pregnancy [16], [17]. In pre-eclampsia however plasma volume expansion is reduced and plasma volume does not reach the same levels as seen in normotensive pregnancy [18]. Those women destined to develop later preeclampsia lose more of a given sodium load then those with a regular pregnancy outcome suggesting a beneficial effect of limiting excess natriuresis [19].

## 2 Salt-sensing: the role of the immune system

The observation in astronauts on board the MIR space station of sodium storage in the skin without accompanying water retention has led the way in extrarenal sodium storage being accepted as part of normal healthy physiology [20], [21], [22]. Furthermore evidence has shown that blood pressure regulation can be influenced by the sodium retained in the subdermal interstitium by mechanisms which involve the immune system. The first of these mechanisms is via upregulated expression of interleukin 17 (IL-17) which has been shown to increase the formation of reactive oxygen species and inflammatory leucocytes within the circulation resulting in endothelial dysfunction and raised systolic blood pressure. Sodium is implicated for this process as high salt concentrations stimulate the development of IL-17 producing CD4^+^ T helper cells, known as Th17 cells [23]. The mechanism by which salt is able to induce Th17 polarisation by T lymphocytes is via p38/MAPK and NFAT5 as well as serum/glucocorticoid-regulated kinase 1 (SGK-1) dependent signalling [23], [24].

SGK-1 is also important in salt sensing and responding to changes in extracellular Na^+^ [25]. Excess production of glucocorticoid or mineralocorticoid, inflammation and hypertonicity all cause upregulation of SGK-1 and this in turn leads to increased activity of a number of ion channels including the epithelial sodium channel (ENaC) [26]. Excessive expression and activity of SGK-1 promotes vascular remodelling and macrophage activation which results in worsening of the pathophysiological processes associated with hypertension, diabetes, tumour growth and infertility [27], [28]. SGK-1 knockout mice when treated with excess mineralocorticoid and high salt show no progression of hypertension. The exact mechanisms by which SGK-1 is involved in hypertension and cardiovascular disease remains to be fully elucidated however they may involve regulation of the transcription factors forkhead transcription factor 3a, beta-catenin and nuclear factor κ B [29], [30], [31].

Tissue macrophages are also important in extrarenal sodium mediated regulation of blood pressure. Following a high salt diet excess Na^+^ is bound in an osmotically inactive form by negatively charged polyanionic glycosaminoglycans present in the skin interstitium. Macrophages actively migrate to areas of hypertonic sodium storage, leading to the suggestion that these cells are mobile osmoreceptors [32]. High levels of sodium result in macrophage expression of tonicity-responsive enhancer binding protein (TonEBP). TonEBP is an osmosensitive transcription factor with a wide range of roles. One of the key roles of TonEBP with regard to blood pressure regulation is that it is able to bind to two sites within the vascular endothelial growth factor C (VEGF-C) promoter and is thus able to regulate VEGF-C expression. VEGF-C released by the macrophages results in increased lymphangiogenesis of pre-existing lymph capillaries by binding VEGFR3 receptors. This enhances the lymph-capillary network in the skin facilitating the clearance of salt [33]. Release of VEGF-C triggers increased endothelial nitric oxide synthase (eNOS) expression via activation of VEGFR2 receptors. The potent vasodilator activity of nitric oxide produced in this manner acts to compensate blood pressure following a high salt diet. VEGF-C release is also able to stimulate induction of Th17 lymphocytes [34]. Reduction in VEGF-C availability via either reduced macrophage numbers or binding to its soluble receptor VEGFR3 leads to augmented interstitial hypertonic volume retention resulting in decreased eNOS expression culminating in increased blood pressure [33]. Intradermal sodium sensing is not suggested to overrule renal regulatory function but rather act in a complementary manner to assist with electrolyte balance, volume regulation and blood pressure control [35].

Salt is also able to regulate the switching of macrophages between their M1 pro-inflammatory and M2 anti-inflammatory phenotypes via salt-inducible kinase (SIK) activity. Inhibition of SIKs promotes an M2 phenotype via IL-10 production. A similar induction of an anti-inflammatory phenotype is also observed in SIK inhibitor treated dendritic cells. The role of SIK in regulating pro-inflammatory and anti-inflammatory cell lineage is limited only to myeloid derived cells and it is unable to influence T lymphocyte development due its signalling pathway which is regulated by MAP kinase pathways rather than the PKA pathways involved in myeloid cell differentiation.

## 3 The importance of endogenous ouabain

The molecular mechanisms by which salt induces the changes that lead to hypertension are unknown. Furthermore the ability of salt to alter blood pressure varies between individuals and is referred to as salt-sensitivity. One candidate molecule which may have a key role in linking salt with hypertension is endogenous ouabain (EO). In high salt intake of salt-sensitive individuals this endogenous cardiotonic steroid is secreted by the adrenal glands and the hypothalamus following elevation of plasma and cerebrospinal fluid Na^+^. It is this salt induced secretion of EO that is believed to be central to salt induced hypertension. The relationship of EO with Na^+^ is complex but both correlate with blood pressure [36]. The way in which EO is able to regulate blood pressure is due to its ability to function as a Na^+^ pump inhibitor thereby preventing renal Na^+^ reabsorption, to enhance vascular tone and regulate Ca^2+^ signalling in myocytes, endothelial cells and neurons [37], [38], [39]. EO binds to Na^+^ pump receptors in both the central nervous system and the periphery, with mutations in these receptors preventing EO-induced hypertension by reducing the binding affinity of EO for these receptors. In patients with essential hypertension EO is found to accumulate in those tissues responsible for blood pressure regulation (e.g kidneys, hypothalamus and posterior pituitary).

Normal pregnancy is an EO- resistant state, where blood pressure remains low despite elevated circulating levels of EO [40]. Moreover, pre-eclampsia and pregnancy induced hypertension are associated with a loss of this resistance to EO where circulating cardiotonic steroids are elevated. Interestingly EO and its high affinity α2 Na^+^ pump receptor are implicated in regulating the return of blood pressure to pre-pregnancy levels during the third trimester. In pregnant α2 Na^+^ pump receptor knockout mice this rise in blood pressure during the third trimester has been shown to be reduced [41].

## 4 The importance of salt for healthy pregnancy

In order for the essential hemodynamic changes to occur that are required during pregnancy and also to maintain blood pressure an adequate salt intake is required during gestation [42].

Studies have also shown that salt restriction is of no benefit to women with hypertensive disorders of pregnancy [43]. A very early intervention study looking at the effect of salt supplementation on the incidence of pre-eclampsia indeed showed that there was a decrease in the incidence of pre-eclampsia, oedema, perinatal death, antepartum haemorrhage and bleeding during pregnancy in women told to increase their salt intake [44]. Furthermore rodent studies utilising a reduced uterine perfusion pressure (RUPP) model have shown that a low salt diet during pregnancy increased arterial blood pressure and vascular reactivity suggesting that this was due to increased Ca^2+^ entry from the extracellular space [45]. Salt restriction during pregnancy is also not recommended as it has also been associated with low nephron number in the offspring [46]. An aldosterone synthase knockout mouse model resulted in a lower number of offspring and IUGR, a phenotype rescued upon high salt intake [47]. Currently there are no recommended guidelines for salt intake during pregnancy and further work is needed to allow the determination of such recommendations.

## 5 Could salt improve the utero-placental immune environment in pre-eclampsia?

Leucocytes represent an important population within the cells present in the placental bed and they have distinctive roles in regulating extravillous cytototrophoblast invasion and spiral artery remodelling [48]. Pre-eclampsia is characterised by a reduction in the number of CD14^+^ macrophages in the placental bed and it is suggested that this culminates in an unfavourable cytokine balance resulting in defective placentation characteristic of pre-eclampsia [49]. Within the skin, interstitial salt is known to function as a chemo-attractant for macrophages. Salt supplementation during early pregnancy is therefore suggested as a potential therapeutic intervention for preventing pre-eclampsia with the aim of restoring CD14^+^ macrophage numbers to the levels seen in normal pregnancy and therefore ensuring normal placental development and function. Although in the non-pregnant situation salt supplementation stimulates IL-17 production, SGK-1 activity and SIK which are all associated with the generation of a proinflammatory environment it is suggested that in pregnancy ensuring that macrophage numbers are adequate by providing salt supplementation will ensure macrophage driven production of transforming growth factor beta (TGFβ) and tumour necrosis factor (TNFα) which are needed to regulate extravillous cytotrophoblast invasion and also ensure adequate production of the vasodilator nitric oxide.

## 6 The placenta as a salt-sensing organ

Both VEGF-C and TonEBP have been shown to be expressed in placental villous and extravillous trophoblast cells and also placental endothelial cells [50]. Expression of both of these was increased following osmotic challenge with sodium chloride, thus indicating that these factors are present and also may be important in salt sensing within the placenta (unpublished data from our own group using an in vivo rodent model). Moreover, it is also known that the placenta contains high levels of proteoglycans [51] which within the skin interstitium have been shown to be able to bind and to accumulate Na^+^ without concomitant water retention. The expression of a number of placental proteoglycans including syndecan-2, glypicans 1 and 3, decorin and perlecan have all been found to be reduced in pre-eclampsia [52]. This evidence therefore indicates that all of the essential components needed for salt sensing, as reported for salt sensing in the skin, are indeed present in the human placenta supporting the concept that the placenta may be playing an important part in the regulation of maternal blood pressure.

As mentioned above SGK1 dependent signalling represents an important way by which salt is able to regulate the immune system via polarising the development of Th17 cells. Expression of SGK-1 has also been confirmed at both the molecular and protein level in decidua with decreased SGK-1 expression being associated with miscarriage [53].

## 7 The importance of aldosterone in pregnancy

Aldosterone, as already described, is important for the plasma volume expansion that occurs during healthy pregnancy and its expression, along with other members of the RAS, is increased in normal pregnancy [54]. In comparison pre-eclampsia is characterised by reduced expression of RAS components and a reduction in plasma volume expansion which is present before the clinical manifestations of pre-eclampsia are apparent. Even though there is intravascular plasma retention as noted by the visible oedema in patients with pre-eclampsia plasma renin activity and aldosterone are in fact reduced compared with normotensive pregnant women [55]. In fact a positive correlation has been shown between fetal birthweight and circulating aldosterone concentrations in women with pre-eclampsia [56].

The regulation of aldosterone during pregnancy is poorly understood. Recent work has shown VEGF to be a more potent inducer of adrenal aldosterone production than angiotensin II [57]. In this study VEGF was shown to regulate both endothelial cell-dependent and independent activation of aldosterone. Furthermore a rodent model showed that the VEGF inhibitor soluble fms-like tyrosine kinase-1 (sFLT-1) resulted in a fall in aldosterone concentrations and it was suggested that this may explain why aldosterone levels are inappropriately low in pre-eclampsia, which is characterised by high levels of sFLT-1, where plasma volume expansion is reduced [57].

Despite already expanded plasma volume expansion during the second trimester of pregnancy aldosterone levels remain high throughout pregnancy and this has led to the suggestion that aldosterone has an additional role in the direct regulation of placental and fetal development [58]. Human first trimester primary trophoblasts, characterised as vimentin and cytokeratin-7 positive, along with trophoblast cell lines JEG-3 and HTR/SV neo all showed a proliferative response following culture with aldosterone showing direct regulation of aldosterone on placental development via activation of mineralocorticoid receptors within the feto-placental unit. Furthermore plasma aldosterone levels taken in the first trimester of pregnancy were found to accurately predict placental weight at term. A murine knockout model has also shown the importance of aldosterone as aldosterone synthase knockout mice have smaller necrotic placentae combined with smaller litter sizes, indicating that aldosterone is important not only in placental growth but also with fetal development [47].

A role for aldosterone in the pathogenesis of pre-eclampsia has also been suggested as a number of studies have reported low aldosterone levels to be characteristic of pre-eclampsia [59]. Reduced aldosterone could offer an explanation for the reduced placental size also found in pre-eclampsia. Loss of function mutations in the aldosterone synthase gene CYP11B2 are found with a high frequency in pre-eclampsia [60] whereas gain of function mutations are found with a low frequency again indicating an important role for aldosterone in normal pregnancy [56].

## 8 Could salt act in the place of reduced aldosterone to maintain blood pressure in pre-eclampsia?

**Fig. 1:**
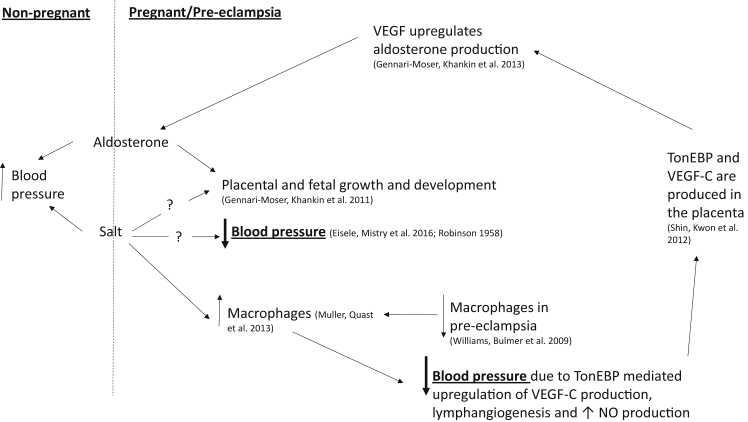
**Overview of potential role for dietary salt supplementation in maintaining healthy blood pressure in pregnancy.** Outside of pregnancy excessive salt consumption is linked with hypertension, however in pregnancy salt has been shown to lower blood pressure, by as yet, unknown mechanisms. Salt may also be able to compensate for aldosterone deficiency via upregulation of macrophages leading to vascular endothelial growth factor (VEGF) production. (TonEBP = tonicity-responsive enhancer binding protein; NO = nitric oxide).

Recent work has now begun to investigate whether salt supplementation may actually be beneficial in pregnancy with particular regard to stimulating the plasma volume expansion needed which does not occur in pre-eclampsia due to low aldosterone production (see Fig. 1). In a case report of a 33 year old woman who had a homozygous loss of function mutation in CYP11B2 it was found that dietary salt supplementation was found to lower blood pressure and keep blood pressure regulated throughout pregnancy leading the authors to suggest that it may be beneficial to offer salt supplementation to pregnant women who show signs of volume deficiency due to reduced levels of aldosterone production [61]. A later study by this same group added further support for the benefits of salt to reduce blood pressure during pregnancy [8]. This study showed that pregnant women spontaneously have a higher salt intake as judged by urinary Na^+^ excretion compared with non-pregnant women. Furthermore pregnant women when given a high salt supplemented diet continued to consume higher levels of salt showing that during pregnancy women can cope with much higher levels of salt consumption than non-pregnant women. What was most interesting in this study was that despite having higher baseline salt intakes the blood pressure of pregnant women dropped and was lower than in non-pregnant individuals. This was combined with the increased occurrence of rising blood pressure in the high salt diet supplementation non-pregnant group.

A murine knockout model has also shown the importance of salt supplementation as a means of compensating for aldosterone deficiency. Aldosterone synthase knockout mice have smaller necrotic placentae combined with smaller litter sizes, indicating that aldosterone is important not only in placental growth but also with fetal development [47]. When these same mice are given a 5% NaCl diet foetal weight is increased and blood pressure falls. This same response of increased foetal weight is also seen in the wildtype animals indicating that dietary salt supplementation during apparently healthy pregnancy may not be harmful. This reduction in blood pressure following salt supplementation has also been replicated in a rodent model which showed significant reductions in systolic blood pressure following high salt diet [62].

## 9 Future perspectives

We are at an exciting time for the possibility of finding a simple dietary supplement that may be able to help alleviate the hypertension associated with pre-eclampsia, however there is still much research to be done to fully elucidate the mechanisms behind this to ensure that the health of both the mother and her baby are not compromised (see Fig. 1). Part of this work will be to fully describe how salt influences the immune system within the placenta and whether salt supplementation is able to help enhance placental bed CD14^+^ macrophage numbers which would be beneficial in pre-eclampsia and whether the placenta is able to function as a salt sensing organ. Further work will also need to identify how salt appears to be able to reduce blood pressure in pregnancy and the involvement of aldosterone.

## Funding

This work was supported by The British Heart Foundation, Basic Science Intermediate Fellowship (FS/15/32/31604) and Swiss National Foundation (3200B0-113902/1, 32-135596).

## Conflict of interest

The authors declare that they have no conflicts of interest related to this review.
